# How Effectively Can Oxidative Stress and Inflammation Be Reversed When CFTR Function Is Pharmacologically Improved?

**DOI:** 10.3390/antiox14030310

**Published:** 2025-03-04

**Authors:** Valeria Rachela Villella, Alice Castaldo, Filippo Scialò, Giuseppe Castaldo

**Affiliations:** 1 CEINGE-Biotecnologie Avanzate Franco Salvatore, 80145 Naples, Italy; valeria.villella@gmail.com (V.R.V.); giuseppe.castaldo@unina.it (G.C.); 2 SC di Pneumologia e UTSIR, AORN Santobono-Pausilipon, 80122 Naples, Italy; alice.castaldo@unina.it; 3 Dipartimento di Scienze Mediche Traslazionali, Sezione di Pediatria, Università di Napoli Federico II, 80131 Naples, Italy; 4 Dipartimento di Medicina Molecolare e Biotecnologie Mediche, Università di Napoli Federico II, 80131 Naples, Italy

**Keywords:** cystic fibrosis, CFTR, inflammation, oxidative stress, ETI

## Abstract

A critical challenge in the age of advanced modulator therapies is to understand and determine how effectively chronic oxidative stress and oxidative stress-induced inflammation can be reversed and physiological balance restored when CFTR function is pharmacologically improved. The triple therapy with elexacaftor–tezacaftor–ivacaftor (ETI) suggests that CFTR activity in individuals with at least one F508del mutation can be partially restored to about 50% of normal levels. Although incomplete, the partial recovery of CFTR function has been shown to drastically lower sputum pathogen content, enhance microbiome diversity, and lower inflammation markers within the first year of treatment in adolescents and adults with cystic fibrosis. However, despite these advancements, residual airway infection, oxidative stress and inflammation persist, with levels similar to other chronic lung conditions, like non-CF bronchiectasis. This persistence highlights the necessity for innovative antioxidant and anti-inflammatory treatments, in particular for individuals with advanced lung disease. To address this issue, emerging multi-omics technologies offer valuable tools to investigate the impact of modulator therapies on various molecular pathways. By analyzing changes in gene expression, epigenetic modifications, protein profiles and metabolic processes in airway-derived samples, it could be possible to uncover the mechanisms driving persistent oxidative stress and inflammation. These insights could pave the way for identifying new therapeutic targets to fully restore airway health and overall physiological balance.

## 1. Introduction

### 1.1. Cystic Fibrosis: An Overview

**Figure 1:**
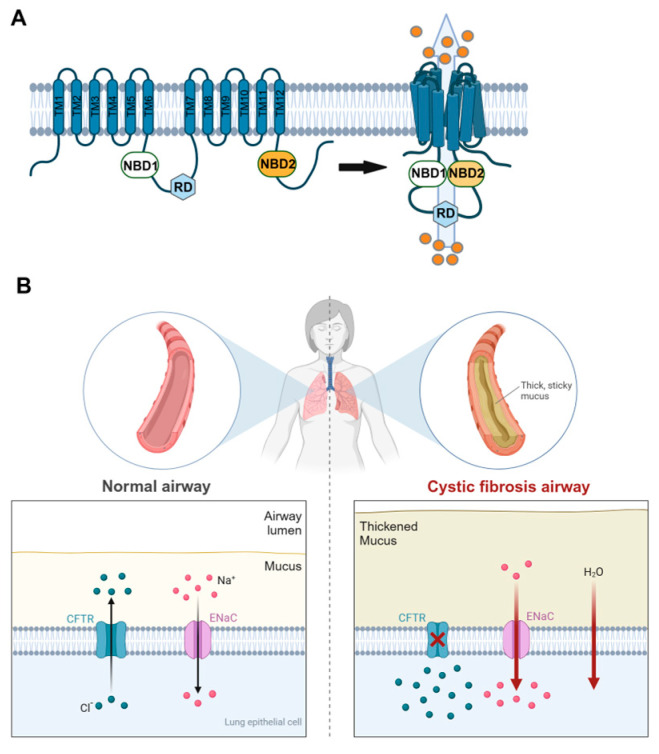
*CFTR* dysfunction in CF. (**A**) The *CFTR* protein consists of five distinct domains. These include two membrane-spanning domains each containing six transmembrane segments (TM1–6 and TM7–12) that together form the channel pore. There are two cytosolic nucleotide-binding domains (NBD1 and NBD2) and a regulatory domain (RD). These five domains are arranged into a compact configuration that transports chloride ions out of the cell. (**B**) CFTR dysfunction leads to chloride ions becoming trapped within the cell, while hyperactive ENaC promotes excessive fluid absorption. This combination depletes the ASL, resulting in a thickened mucus layer and compressed cilia, which ultimately impairs mucociliary clearance. Image produced by biorender.com.

**Figure 2:**
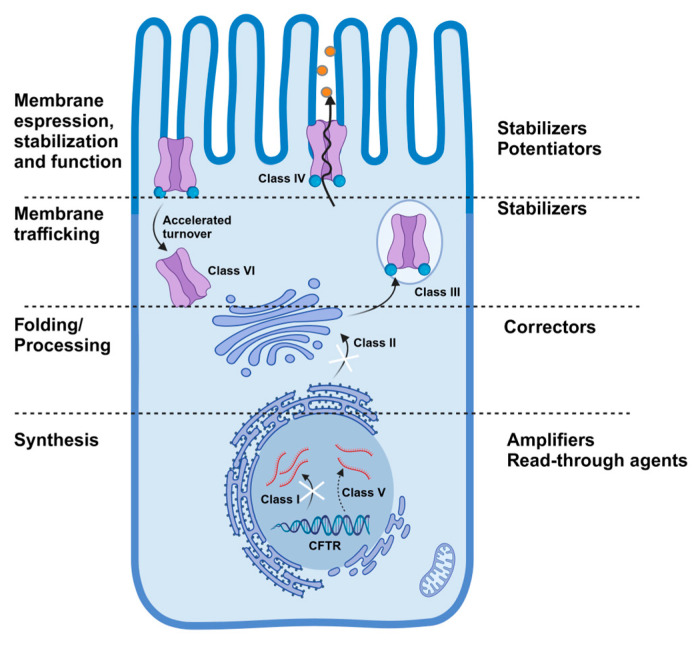
*CFTR* classes of mutations. Class I mutations cause the introduction of premature stop codons. Class II mutations impede both the folding and trafficking of CFTR. Class III mutations cause defects in channel opening. Class IV causes reduced chloride transport. Class V causes limited protein production. Class VI causes decreased stability. CFTR dysfunctions cause the accumulation of thick that restricts the airflow creating the conditions for bacterial colonization, airway infection and inflammation which result in lung damage. Image produced by biorender.com.

Advancements in treatments for cystic fibrosis (CF) have been driven by identifying its genetic origin: mutations in the *CFTR* gene. This gene encodes an ion channel responsible for transporting chloride and bicarbonate across the epithelial cell membrane. The CFTR protein’s activity is vital for maintaining fluid and ion balance in the airways, pancreas, gastrointestinal system and sweat glands [1,2]. The CFTR protein structure consists of two transmembrane domains, two nucleotide-binding regions and a regulatory domain (Figure 1). This protein is activated through phosphorylation by protein kinase A, enabling it to transport chloride and bicarbonate ions. This transport regulates the volume, pH and salt levels of epithelial surfaces, playing a critical role in maintaining cellular homeostasis [3,4,5]. More than 2000 mutations have been identified in the *CFTR* gene, with around 700 confirmed to cause CF. These mutations disrupt CFTR functionality through several mechanisms. Class I mutations, such as nonsense and frameshift alterations, prevent protein synthesis by introducing premature stop codons. Class II mutations hinder the proper folding and trafficking of CFTR, resulting in little or no functional protein reaching the cell surface. Mutations in the *CFTR* gene can also include defects in channel opening (class III), reduced ion conductance (class IV), limited protein production (class V) and decreased stability (class VI) (Figure 2) [6,7]. Among these, the most prevalent mutation, a deletion of phenylalanine in position 508 (F508del), affects about 85% of CF patients and is classified as a class II defect. This mutation results in minimal CFTR channels reaching the cell membrane due to issues in protein folding and trafficking [8] (Table 1). Modern genomic and population genetic studies have greatly enhanced our understanding of the ancestral origins of *CFTR* mutations. The F508 mutation is primarily associated with European origins, while G551D and W1282X have been linked to specific ethnic populations (Celtic and Ashkenazi Jewish, respectively). These insights not only help trace the evolutionary history of cystic fibrosis, but also inform current clinical and genetic counseling practices by highlighting the impact of historical migration and founder effects [9,10,11,12].

Although the pathophysiological consequences of CFTR dysfunction are systemic, notably affecting various organs [13,14,15], the main consequences can be seen in the lungs, where the accumulation of thick, sticky mucus that clogs the airways, restricts airflow and creates an environment for persistent airway infection and inflammation, leading to progressive lung damage. Several mechanisms have been demonstrated to contribute to the abnormal mucus properties and impaired mucociliary clearance in CF. Many studies have shown that an imbalance between CFTR-mediated secretion and ENaC-mediated absorption results in excessive fluid absorption, which depletes the airway surface liquid (ASL) and causes the mucus layer to become highly concentrated (Figure 1) [16,17]. This, in turn, compresses the underlying cilia, impairing their ability to clear the mucus. Moreover, the oxidant acids generated by neutrophil peroxidases increase disulfide crosslinking in the large mucins MUC5B and MUC5AC, resulting in greater viscoelasticity and dysfunction of the mucus gel [18]. Additionally, has been demonstrated that CFTR dysfunction lowers the pH of the ASL, reducing antimicrobial peptide activity and increasing vulnerability to infections of well-known CF pathogens such as staphylococcus aureus (Sa), pseudomonas aeruginosa (Pa) and hemophilus influenzae (He) [19,20]. Together, these findings highlight how CFTR dysfunction disrupts normal mucociliary function and host defenses, perpetuating the cycle of infection and inflammation in CF airways.

### 1.2. Pharmacological Strategies for Restoring *CFTR* Function

Understanding the molecular basis of *CFTR* mutations has been critical in shaping treatment strategies, as it became evident that different mutations require tailored therapeutic approaches [21]. The F508del mutation, for example, was found to cause two specific defects: instability in the nucleotide-binding domain 1 and improper interdomain assembly. Therefore, it was clear that both problems had to be addressed simultaneously to achieve effective restoration of CFTR function [22,23]. Moreover, it is increasingly recognized that single mutations often cause multiple functional impairments. The F508del mutation not only disrupts protein folding and trafficking, but also affects channel gating and reduces stability at the cell surface. Other mutations, such as W1282X, N1303K and R117H, exhibit a combination of defects, further complicating treatment strategies [24]. This complexity may in part explain why responses to pharmacological treatments vary among patients, even for mutations within the same class.

The development of CFTR modulator therapies, named highly effective modulator therapy (HEMT) [25], has revolutionized CF treatment by addressing both the folding and gating defects in mutant CFTR channels through a combination of corrector and potentiator agents (Figure 2) [26]. This has resulted in remarkable improvements in lung function and clinical outcomes for eligible patients, thus improving their clinical management. The current triple combination including two different correctors—elexacaftor (a type III corrector) and tezacaftor (a type I corrector)—paired with the potentiator ivacaftor (ETI) is the most studied. This combination has shown the greatest effectiveness in patients, with at least one F508del mutation restoring CFTR function to around 50% of normal CFTR activity [27,28,29,30,31,32,33]. In addition, 177 other rare *CFTR* variants have been identified to respond positively to ETI, allowing its approval for use in patients with these specific variants by the US Food and Drug Administration (FDA) [34]. As a result, more than 90% of cystic fibrosis patients are now genetically eligible for modulator therapies, and the list of CFTR variants responsive to ETI is continuously expanding through patient-derived models, such as intestinal organoids [35,36] and airway epithelial cells [37,38] which closely mimic native patient tissues [39,40,41]. These models play a crucial role in personalized treatment by predicting therapy responses for patients with rare mutations, enabling treatment optimization as more CFTR-modulating drugs are becoming available. Continuous improvement is being pursued with the development of more efficient and stable correctors, such as vanzacaftor and the potentiator deutivacaftor, with the aim of potentially reducing adverse events and off-target effects that we and others have demonstrated for some ETI components such as tezacaftor and ivacaftor [42,43,44]. In patients with at least one F508del allele, the RIDGELINE and SKYLINE phase 3 trials have shown that once-daily dosing of the triple combination therapy vanzacaftor–tezacaftor–deutivacaftor is both safe and well-tolerated in CF patients aged 6–11 years, as well as in individuals aged 12 years and older [45,46]. Furthermore, the predicted FEV1% and sweat chloride levels were similar to those anticipated with ETI therapy. It is important to state that while the vanzacaftor–tezacaftor–deutivacaftor therapy offers significant clinical benefits for individuals with CF, its global accessibility remains uneven. High-income regions benefit from quicker regulatory approvals and reimbursement support, whereas economic constraints, regulatory delays and limited distribution infrastructures challenge availability in many low- and middle-income countries [47]. Additionally, current approvals generally cover patients aged 6 years and older, with ongoing research needed to explore the safety and efficacy of this therapy in younger pediatric populations [45,46]. Efforts to address these challenges are crucial for ensuring equitable access to this life-changing therapy worldwide.

The remaining 10% of CF patients are affected by class I mutations which cause the block of full-length CFTR protein production (Figure 1). Promising treatments include the use of aminoglycoside analogs ELX-02, a readthrough agent currently in phase 2 trials, and SRI-41315, which boosts *CFTR* expression and enhances aminoglycoside effects [48]. The inhibition of nonsense-mediated mRNA decay (NMD) with SMG1i also provides an alternative strategy to further improve the efficacy of readthrough agent outcomes [49]. The combination of these new therapeutic approaches with CFTR modulators, like ETI, has shown superior CFTR restoration, offering hope for patients with class I mutations.

## 2. Oxidative Stress in Cystic Fibrosis

**Figure 3:**
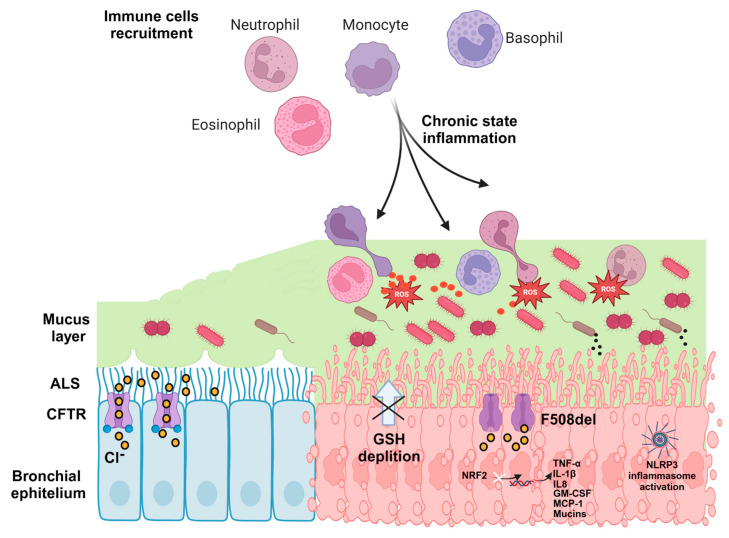
Oxidative stress and inflammation in CF. The impaired mucociliary clearances cause a persistent bacterial infection. This promotes epithelial cells to release pro-inflammatory cytokines, which recruit neutrophils to the infection site. Consequently, a self-perpetuating cycle of neutrophilic inflammation and oxidative stress develops, driven by the excessive production of ROS from epithelial cells and neutrophils. This cycle results in irreversible airway destruction and fibrosis. Additionally, reduced GSH levels exacerbate oxidative stress. Image produced by biorender.com.

As discussed previously, the defective CFTR protein impairs mucociliary clearance, leading to mucus accumulation and creating a niche for bacterial pathogens like Sa, Pa and Hi. The immune system responds with an influx of neutrophils which release both proteases and reactive oxygen species (ROS) as part of the respiratory burst aimed at destroying these pathogens. However, the excessive and prolonged production of ROS overwhelms the antioxidant defenses, resulting in oxidative stress (OS) and tissue damage [50]. Moreover, CFTR dysfunction impairs the export of glutathione (GSH) from the cell, thereby heightening cellular vulnerability to oxidative damage and apoptosis (Figure 3) [51]. The essential role of GSH in maintaining cellular redox homeostasis and acting as a key antioxidant that safeguards lung tissue against oxidative injury has been extensively documented in various lung pathologies [52,53,54]. It is well established that individuals with CF have reduced GSH levels with a consequent disruption in the extracellular balance with a marked increase in the oxidized GSH species [55,56,57]. Consequently, the ability to counteract OS triggered by neutrophils during infections is significantly impaired. Moreover, ROS generated by neutrophils, such as hypochlorous acid produced by myeloperoxidase (MPO), further oxidize GSH, perpetuating a damaging cycle (Figure 3). The inhibition of MPO with AZM198 reduced OS in a mouse model of CF [58]. Despite the high relevance of targeting this pathway, clinical investigation on the benefit of GSH supplementation has remained limited. The diminished antioxidant response is also exacerbated by the dysfunction of the Nrf2 pathway [59,60], a critical transcription factor that regulates the expression of over 200 antioxidant and detoxification genes, including heme oxygenase-1 (HO-1), NAD(P)H quinone oxidoreductase 1 and glutamate-cysteine ligase. In CF patients, the insufficient activation of the HO-1/carbon monoxide (CO) pathway in monocytes and macrophages leads to an unbalanced immune response and defective bacterial killing [61], resulting in an excessive inflammation. Furthermore, in addition to the robust production of ROS from neutrophils, pathogens like Pa can produce toxins such as pycocyanin, which has been shown to interfere with cellular redox balance and generate ROS, further exacerbating OS in CF patients [62]. Lipid metabolism abnormalities also play a role in the link between CFTR dysfunction and OS. Alterations in fatty acids, ceramides and cholesterol metabolism contribute to chronic airway damage in CF [63,64]. Increased ceramide levels, sphingosine phosphate (S1P) dysregulation and reduced lipid catabolism are associated with enhanced inflammation, OS and defective autophagy [65]. Modulating lipid synthesis, such as through sphingolipid inhibitors like myrocin, has been shown to decrease inflammation, restore fatty acid oxidation and improve pathogen clearance in CF models [66].

Although HEMT can improve many aspects of the CF pathology [25], it does not fully restore a normal redox balance. Adjuvant therapeutic approach to counterbalance OS, in conjunction with HEMT treatment, includes the use of N-acetylcysteine (NAC), an antioxidant that can help replenish GSH [67,68]. NAC has been used in CF patients as a mucolytic and as an antioxidant; however, its benefits for OS specifically in CF are still being studied. As natural antioxidants, vitamin E and vitamin C are part of supportive treatment strategies, and several studies have shown mixed results in terms of their direct effectiveness for CF lung disease [69,70,71]. More focus was placed inhaled GSH as a therapy to improve lung function. Many studies have been conducted to verify the effects of inhaled GSH, but have found modest benefits in the improvement of lung function and potential reductions in OS [72]. However, as with many studies, the degree of benefit may vary depending on individual patient characteristics, the stage of the disease and other treatment factors [73].

## 3. Inflammation in Cystic Fibrosis

It is well known that individuals with CF show elevated levels of inflammation. Interestingly, already in the 1990s, many clinical studies were able to detect high levels of inflammation in the first few weeks of life [74]. Bronchoalveolar lavage fluid (BALF) from 4 weeks old CF children showed that this inflammation was characterized by increased neutrophils and macrophage recruitment, large amounts of active elastase and pro-inflammatory molecules such as interleukin (IL) 8, IL6 and tumor necrosis factor alpha (TNF-α) [75]. Interestingly, these inflammatory markers were observed even in the absence of detectable bacterial infections [74,75]. This observation suggests that, in people with CF, inflammation initiates before microbial infections and is triggered as a response to the misfolded CFTR protein in epithelial cells [76]. Generally, the inflammation mechanism in CF patients is multifaceted, involving multiple immune responses and cellular processes. Several immune mediators such as the pro-inflammatory cytokines IL-8, IL-6 and TNF-α, together with the bacterial chemoattractant, have been demonstrated to cause the persistent influx of polymorphonuclear neutrophils, which release various harmful substances, including ROS and proteolytic enzymes, causing damage to surrounding tissues [77]. IL-8, the primary neutrophil chemoattractant, is elevated in the sputum, bronchoalveolar lavage fluid (BALF) and serum of CF patients, even during stable clinical periods. These increased levels correlate with the number of neutrophils present in the BALF [78]. Moreover, in CF patients, an excessive release of neutrophil extracellular traps (NETs) have been shown to contribute to lung damage by promoting fibrosis due to the accumulation of inflammatory debris [79]. Finally, as these neutrophils break down, they release DNA, which contributes to the thick and sticky consistency of CF mucus. During lung infection flare-ups, the DNA levels in the sputum rise, primarily from human sources rather than bacteria [80].

An emerging theory suggests that the dysregulation of immune cells is a central driver of the excessive lung inflammation observed in cystic fibrosis, independent of external factors such as bacterial infections, oxygen deficiency and pH alterations. Two different lines of evidence exist in the literature. The first supports the idea that CFTR loss of function in lymphocytes, neutrophils, monocytes and macrophages is associated with delayed or altered resolution of lung inflammation [81,82]. Bone marrow transplant experiments between wild-type and CFTR-deficient mice indicate that the heightened pro-inflammatory cytokine levels in CF lungs result from CFTR deficiency in immune cells, particularly macrophages, rather than epithelial cells [83]. The second theory suggests that monocytes from CF patients display an altered DNA methylation profile that correlates with a hyperinflammatory state, suggesting that epigenetic reprogramming drives their dysregulated phenotype [84]. Additionally, distinct histone modifications in macrophages are associated with an increased expression of pro-inflammatory genes [85], indicating that epigenetic mechanisms may contribute to the altered innate immune responses in CF individuals.

Since macrophages are vital for initiating and resolving inflammation, their dysfunction may drive early and progressive CF lung disease. The initial inflammatory reaction caused by CFTR malfunction or altered epigenetic modifications is then intensified by bacterial colonization, as microbes accumulate on the dense mucus layer formed due to decreased ASL and compromised mucociliary clearance. This leads to persistent bacterial infections, which act as a continuous trigger for an unrelenting inflammatory response. Over time, the inability to regulate inflammation response leads to continued tissue damage, formation of a thick inflammatory exudate that infiltrates the submucosa extending to the airway wall and surrounding structures, ultimately resulting in bronchiectasis [86]. The inflammatory masses progressively replace the airways, resulting in permanent injury, progressive lung deterioration and eventually results in fatality.

## 4. CFTR Modulator Therapy Cannot Resolve Infection

The primary clinical characteristics of CF lung disease include chronic airway infections and frequent acute exacerbations, contributing to irreversible lung damage. The key pathogens that chronically infect the airways in CF are Sa, Hi, Burkholderia cepacian (Bc) and Pa. Sa and Hi typically colonize the CF airways early, often before clinical symptoms appear, while Pa infection almost always occurs after the other pathogens are present [87]. The relationship between lung dysbiosis and CF is complex. While microbial composition can vary widely among CF individuals, research suggests that a more diverse microbial community in the lungs correlates with better overall lung health [88]. Reduced microbial diversity is often associated with more severe airway inflammation and greater lung function deterioration. Recent studies [89] have shown that restoring a healthy microbial balance, or at least minimizing harmful microbial overgrowth, could play a role in managing CF-related lung disease. The researchers analyzed sputum samples and measured pro-inflammatory cytokines (IL-1β, IL-8, IL-6, TNF-α) along with neutrophil elastase (NE) levels. The results showed that greater microbial diversity (α-diversity) in the airways was associated with lower inflammation and better lung function, as reflected by higher FEV1% predicted. The current therapy to resolve infection for CF patients includes the use of antibiotics (oral, inhaled or intravenous), mucolytics and hydrators, bronchoscopy with lavage (for mucus plugs or severe infections) or the use of emerging adjunctive therapies, such as bacteriophage therapy [90]. In this context, CFTR modulator therapies have been shown to significantly improve lung function and reduce pulmonary exacerbations in CF patients. The primary effects of modulators therapy were: (i) improvement mucus hydration and clearance, reducing bacterial colonization; (ii) improving airway microbial diversity, often leading to a shift toward a less pathogenic microbiota; (iii) reduction in levels of inflammatory markers, like IL-8 and NE. Patients with chronic infections (e.g., Pa and Bc) may see a reduced bacterial load, but often do not achieve complete eradication. Some studies suggest that modulators may improve antibiotic effectiveness by enhancing drug penetration into airway secretions [91]. While modulators improve lung function and reduce infections, they do not completely eliminate lung pathogens [92]. An exhaustive review of Saluzzo et al. [93] critically examines current evidence regarding the impact of CFTR modulators on airway infections by highlighting that current research is exploring the impact of CFTR modulator therapies on reducing chronic and recurrent airway infections in CF. Early findings indicate that CFTR modulators, especially ivacaftor, may help lower bacterial prevalence, including Pa, though results vary and the long-term benefits remain unclear. Additionally, in vitro studies suggest that some modulators possess direct antimicrobial properties and may enhance antibiotic efficacy; however, further studies are required to confirm these effects.

## 5. CFTR Modulator Therapy Cannot Resolve Inflammation

It is evident that inflammation plays a key role in the pathogenesis of CF and is the focus of major therapeutic research due to its major role in the initiation and progression of lung damage. This focus remains imperative, as emerging evidence shows that inflammation can persist despite the benefits provided by new CFTR modulator therapies. Studies involving ETI showed a decrease in both airway and systemic inflammatory markers 12 months after initiating ETI treatment. However, it is important to note that the levels of these inflammatory markers and proteases did not return to those seen in healthy individuals [94]. In a recent study, these levels were found to be similar to those in patients with non-CF bronchiectasis, a form of chronic lung inflammation used as a control [95]. Another recent study demonstrated that, in CF individuals after 12 months of ETI treatment, there was a reduction in total allergen-specific immunoglobulin E (IgE), but no change in absolute eosinophil count, suggesting that type 2 inflammation is not reduced [96].

For years, research has focused on developing modulators to fix defects in CFTR protein function and trafficking. While these treatments have significantly improved the lives of people with CF, the critical role of inflammation in this condition means that equal effort should be directed toward treatments targeting lung inflammation. Relying solely on corticosteroids and ibuprofen, which provide limited benefits and come with challenges like long-term side effects, is not enough [97,98]. Furthermore, it is uncertain whether these modulators alone can effectively address the inflammation, mucus buildup and lung damage seen in adolescents and adults with severe CF. Similarly, their ability to prevent early lung infection, inflammation, and mucus production in infants showing initial signs of the disease remains unclear. Therefore, there is a critical need for innovative antioxidant and anti-inflammatory therapies that can complement CFTR modulators and antibacterial treatments.

The search for new anti-inflammatory drugs has met numerous obstacles since the primary aim of an inflammatory treatment would be to reduce an excessive inflammatory response without compromising the immune system’s ability to fight infection. This lesson was learned after a phase two clinical trial based on the administration of BIIL 284 BS, a potent inhibitor of neutrophil recruitment, which had to be prematurely stopped due to the high incidence of adverse events [99]. To overcome the strong inhibition of BIIL 284 BS, a more recent phase II trial explored the efficacy of eicosanoid modulators as an anti-inflammatory treatment. This trial tested CTX-4430 (acebilustat), a leukotriene A4 (LTA4) hydroxylase, which reduces the production of leukotriene B4 (LTB4), facilitating a more controlled regulation through careful dosing. This approach avoids the complete suppression of LTB4 activity, reducing the risk of making the host more vulnerable to infections [100]. Another approach could involve harnessing the properties of macrophages by boosting their ability to resolve inflammation and promote tissue repair, functioning as a natural regulator of the inflammatory response. Many studies have demonstrated that the resolution phase of inflammation appears to be impaired in CF [101,102]. Additionally, CFTR dysfunction or altered epigenetic modifications limits the ability of macrophages to shift into an anti-inflammatory, tissue-repairing M2-like state, promoting persistent airway inflammation [103]. A group of interesting targets is represented by the specialized pro-resolving mediators (SPMs), a group of lipid molecules which play a vital role in resolving inflammation by stopping neutrophil activity (e.g., via lipoxin A4 or LXA4) and supporting tissue healing. CF individuals exhibit significantly reduced LXA4 levels and decreased expression of its receptor, ALX/FPR2, in macrophages and epithelial cells with the F508del mutation, linking these deficiencies directly to CFTR malfunction [104]. Recent studies have shown that treating Pa-infected CF mice with resolvin D1 (RvD1), an SPM, significantly reduced bacterial load and neutrophilic inflammation. This treatment also enhanced the clearance of neutrophils/T-cells, improved the ability of CF airway macrophages to kill Pa and lowered the secretion of pro-inflammatory cytokines IL-8 and IL-6 [105]. The NLRP3 inflammasome is also becoming increasingly recognized as a key factor underlying inflammation seen in CF individuals even before infection, as it can be triggered by homeostatic imbalances that are not exclusively pathogen-driven [106]. Various mechanisms have been suggested to induce NLRP3-related inflammation, including shifts in immunometabolism like the Warburg effect, alterations in intracellular ion levels, mitochondrial impairment and endoplasmic reticulum stress [76]. The NLRP3 inhibitor MCC950 has been shown to reduce IL-1β levels in the lungs and improve bacterial clearance in a CF mouse model [107]. Additionally, a recent in vitro study revealed that elevated intracellular Cl^−^ levels, resulting from CFTR dysfunction, drive IL-1β release, a process that is effectively mitigated by MCC950 [108].

## 6. Adjuvant Therapy in Combination with HEMT

More emerging therapeutic approaches are becoming available for CF patients that can act synergistically with HEMT to control the symptoms of disease. Antioxidants are increasingly studied as adjuvant therapy for CF due to their potential to reduce OS, which is heightened in CF patients. Chronic inflammation and recurrent infections lead to excessive ROS production, contributing to lung damage. Antioxidants like vitamins C, E and GSH can help neutralize ROS, protecting cells from oxidative injury. Studies suggest that supplementation may improve lung function and reduce exacerbation. However, the optimal dosage and long-term benefits remain under investigation. Azithromycin (macrolide antibiotic) is commonly used for maintenance therapy in CF patients with chronic Pa infections, helping to improve lung function and reduce exacerbations [109] by showing significant anti-inflammatory and immunomodulatory effects. Acebilustat, an inhibitor of LTA4 hydrolase, reduces neutrophil migration and elastase production [99]. In cannabinoid-derived compounds [110], lenabasum, a selective CB2 receptor agonist, offers anti-inflammatory effects without central nervous side effects. R-roscovitine encourages neutrophil apoptosis, enhances macrophage bactericidal activity and facilitates the trafficking of the F508del to the plasma membrane, alleviating inflammation. Thymosin alpha 1 and anakirna, two naturally occurring immunomodulators, have demonstrated anti-inflammatory properties in CF models by reducing pro-inflammatory cytokines and improving CFTR stability [111,112,113,114].

## 7. Conclusions

In the era of advanced CFTR modulator therapies, significant strides have been made in restoring CFTR function, particularly with triple therapies like elexacaftor–tezacaftor–ivacaftor (ETI). These advancements have improved clinical outcomes for a majority of CF patients. However, residual infection, inflammation and structural lung damage remain persistent challenges, especially in individuals with advanced or longstanding disease. Emerging evidence highlights that while modulators significantly reduce oxidative stress, inflammation and microbial burden, they do not fully restore airway health to pre-disease levels, supporting the need for a specific antioxidant [83] and anti-inflammatory therapy that could complement modulator therapy. A deeper understanding of the molecular pathways underlying persistent airway inflammation, oxidative stress and infection is crucial. Multi-omics approaches provide a promising approach for unraveling these mechanisms, allowing the identification of new therapeutic targets. Strategies such as enhancing oxidative stress defenses, modulating lipid metabolism, targeting macrophage dysfunction and employing SPMs hold significant potential. Furthermore, the development of anti-inflammatory drugs, like NLRP3, inflammasome inhibitors and carefully balanced eicosanoid modulators could represent a vital complement to CFTR modulators.

A multifaceted approach integrating CFTR modulators with targeted antioxidants and anti-inflammatory therapies is essential to achieving the full restoration of airway function and a sustained reduction in lung damage. Future research should prioritize early interventions in pediatric populations, aiming at preventing oxidative stress and inflammation with a consequent delay in irreversible lung injury. By linking the remaining therapeutic gaps, we can further transform the trajectory of CF and improve the quality of life of all affected individuals.

## Figures and Tables

**Table 1:** Summary of the details of each mutation class, including its severity, mutation type, a representative example of the most common mutation, the defect it causes in CFTR processing and the associated pharmacological therapy.

Class of Mutation	Class I	Class II	Class III	Class IV	Class V	Class VI	Class VII
Severity	Severe	Severe	Severe	Mild	Mild	Mild	Severe
Type	Nonsense/ frame-shift	Missense; amino acid deletion	Missense	Missense	Missense splicing defect	Missense	Large deletions
Mutation example	G542X, R553X, R1162X, W1282X	G85E, I507del, F508del, N1303K	S549R, G551D, G1349D	R117H, R347P, R334W, R1070W	A455E, 3272-26A>G	4326del TC, Gln1412X, 4279insA	Dele2,3 (21kb), 1717-1G>A
CFTR defect	No synthesis	Trafficking defect	Impaired channel gaiting	Reduced conductance	Reduced protein	Decreased protein stability	No mRNA
Potential therapy	Read-through agents	Correctors (+ Potentiators) Lumacaftor (+ Ivacaftor)	Potentiators (Ivacaftor)	Potentiators (Ivacaftor)	Splicing modulators amplifiers	Stabilizer	Unrescuable
